# Enhanced *Z*-isomerization of tomato lycopene through the optimal combination of food ingredients

**DOI:** 10.1038/s41598-019-44177-4

**Published:** 2019-05-28

**Authors:** Masaki Honda, Hakuto Kageyama, Takashi Hibino, Ryota Takemura, Motonobu Goto, Tetsuya Fukaya

**Affiliations:** 1 Faculty of Science & Technology, Meijo University, Shiogamaguchi, Tempaku-ku, Nagoya, 468-8502 Japan; 2 Innovation Division, Kagome Company, Limited, Nishitomiyama, Nasushiobara, 329-2762 Japan; 3 Department of Materials Process Engineering, Nagoya University, Furo-cho, Chikusa-ku, Nagoya, 464-8603 Japan; 4 Institutes of Innovation for Future Society, Nagoya University, Furo-cho, Chikusa-ku, Nagoya, 464-8603 Japan

**Keywords:** Biological techniques, Chemical engineering, Nutrition

## Abstract

In tomatoes, most lycopene is present in the all-*E*-configuration and shows very low bioavailability, whereas the *Z*-isomers show higher bioavailability. Hence, for health reasons, it is expected that the ingestion of lycopene *Z*-isomers is preferable. Very recently, it was reported that onion and possibly garlic promoted thermal *Z*-isomerization of (all-*E*)-lycopene but there are no reports for other food ingredients. Here we show new food ingredients that enhance thermal *Z*-isomerization of lycopene in tomatoes and from the results, we guessed some causative components having the *Z*-isomerization promoting effect. A comprehensive investigation of food ingredients revealed that some vegetables (*Allium* sp., *Brassica* sp., and *Raphanus* sp.), shiitake mushroom (*Lentinus edodes*), and some edible seaweeds (*Saccharina* sp. and *Ecklonia* sp.) markedly promoted *Z*-isomerization of (all-*E*)-lycopene in tomato puree with heating at 80 °C for 1 h. Moreover, it was revealed that polysulfides, isothiocyanates, carbon disulfide, and iodine, which were commonly contained in the above food ingredients in considerable quantity, enhanced thermal *Z*-isomerization of (all-*E*)-lycopene. Our findings on the food ingredients and the food-derived catalysts having a carotenoid *Z*-isomerization promoting effect are important, not only for the food, drink, and dietary supplement manufacturing industries, but also for daily home cooking.

## Introduction

Lycopene, an acyclic carotenoid (C_40_H_56_), is a typical functional ingredient contained in tomato (*Solanum lycopersicum*) which is one of the most popular and widely consumed vegetables in the world^1^. Lycopene shows high antioxidant capacity, and the daily consumption of lycopene-rich foods could reduce risk of various diseases such as cardiovascular disease, colorectal adenomas, and prostate cancer^2,3^. However, the absorptivity of lycopene from raw tomatoes and processed tomato products is very low; as an example, Ryan *et al*. (2008)^4^ reported that the bioavailability of lycopene from raw tomato and cooked tomato was less than 10%, which was evaluated by an *in vitro* digestion model. To enhance the bioavailability of lycopene, *Z*-isomerization has recently attracted attention. A number of studies have reported that, in a human dosing test as well as *in vitro* and *in vivo* tests, *Z*-isomers of lycopene showed greater bioavailability than the all-*E*-isomer^5,6^. For example, a human oral administration test showed that *Z*-isomer-rich tangerine tomato juice was more than 8 times more bioavailable than an all-*E*-isomer-rich red tomato juice^5^. Very recently, we reported that olive oil containing 2% garlic extract promoted thermal *Z*-isomerization of (all-*E*)-lycopene and identified diallyl disulfide as a major causative component^7^. Furthermore, de Alvarenga *et al*. (2017)^8^ reported that onion enhanced the production of lycopene *Z*-isomers, whereas they concluded that garlic had no noticeable effect on lycopene *Z*-isomerization^8^. Thus, further investigation is needed to clarify the influence of garlic on the *E*/*Z*-isomerization of (all-*E*)-lycopene. To the best of our knowledge, excluding onion and garlic, there are no reports of food ingredients that promote *Z*-isomerization of (all-*E*)-carotenoids. Therefore, it is important to investigate specific food ingredients and seek to identify components associated with promoting lycopene isomerization for the food, drink, and dietary supplement manufacturing industries as well as for daily home cooking.

## Results and Discussion

### Effect of food ingredients on thermal *Z*-isomerization of tomato lycopene

**Figure 1:**
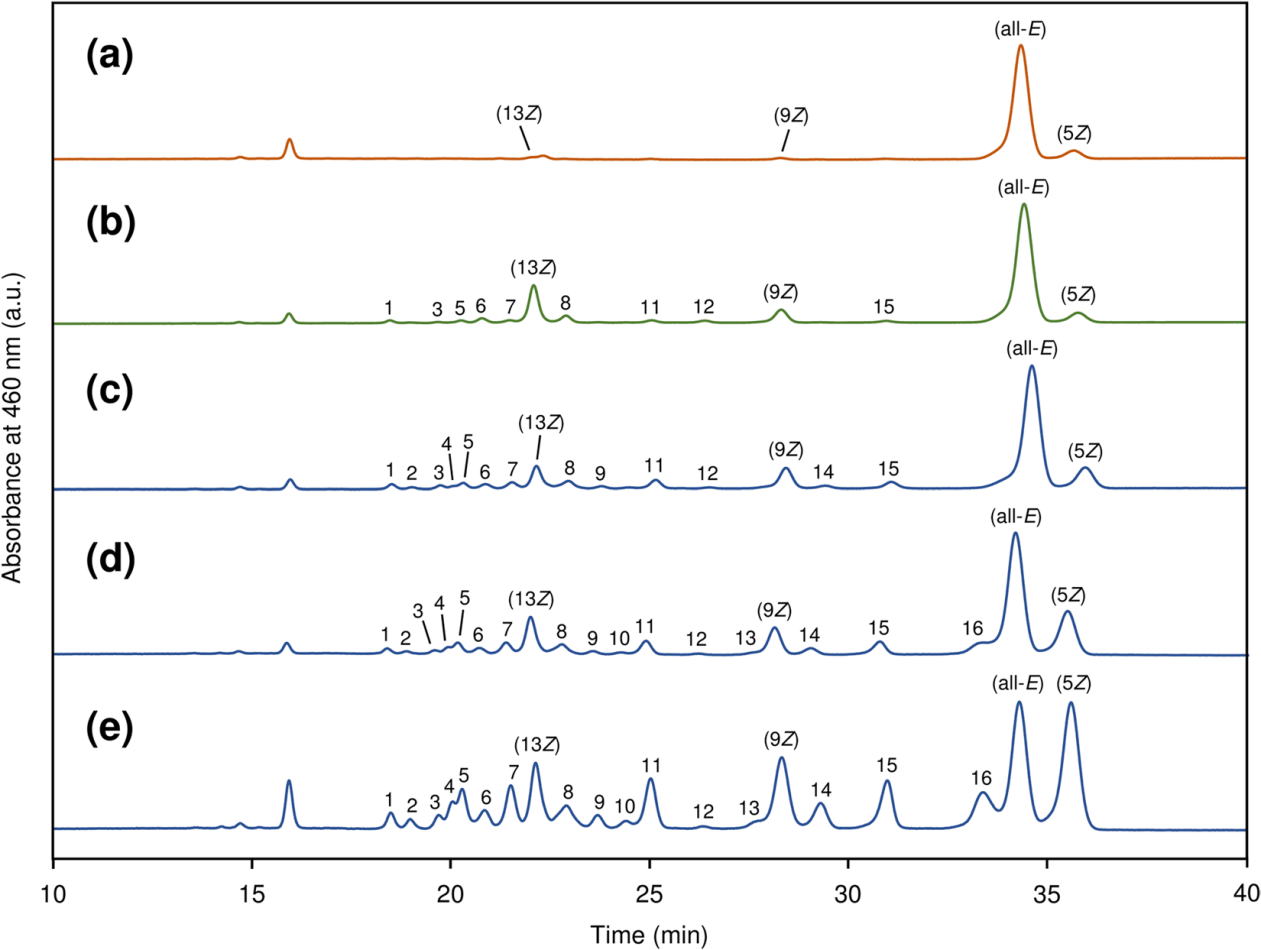
Normal-phase HPLC chromatograms of (**a**) untreated tomato puree and thermally treated tomato puree containing olive oil (**b**) without food ingredient and with (**c**) fresh shiitake mushroom (*Lentinus edodes*), (**d**) fresh garlic (*Allium sativum*), and (**e**) dried ma-kombu (*Saccharina japonica*). (5*Z*)-, (9*Z*)-, and (13*Z*)-Lycopene designated in the chromatograms were identified according to the previous literatures^7,9^. The peaks (1–16) were tentatively identified as shown in Supplementary Table S1.

**Figure 2:**
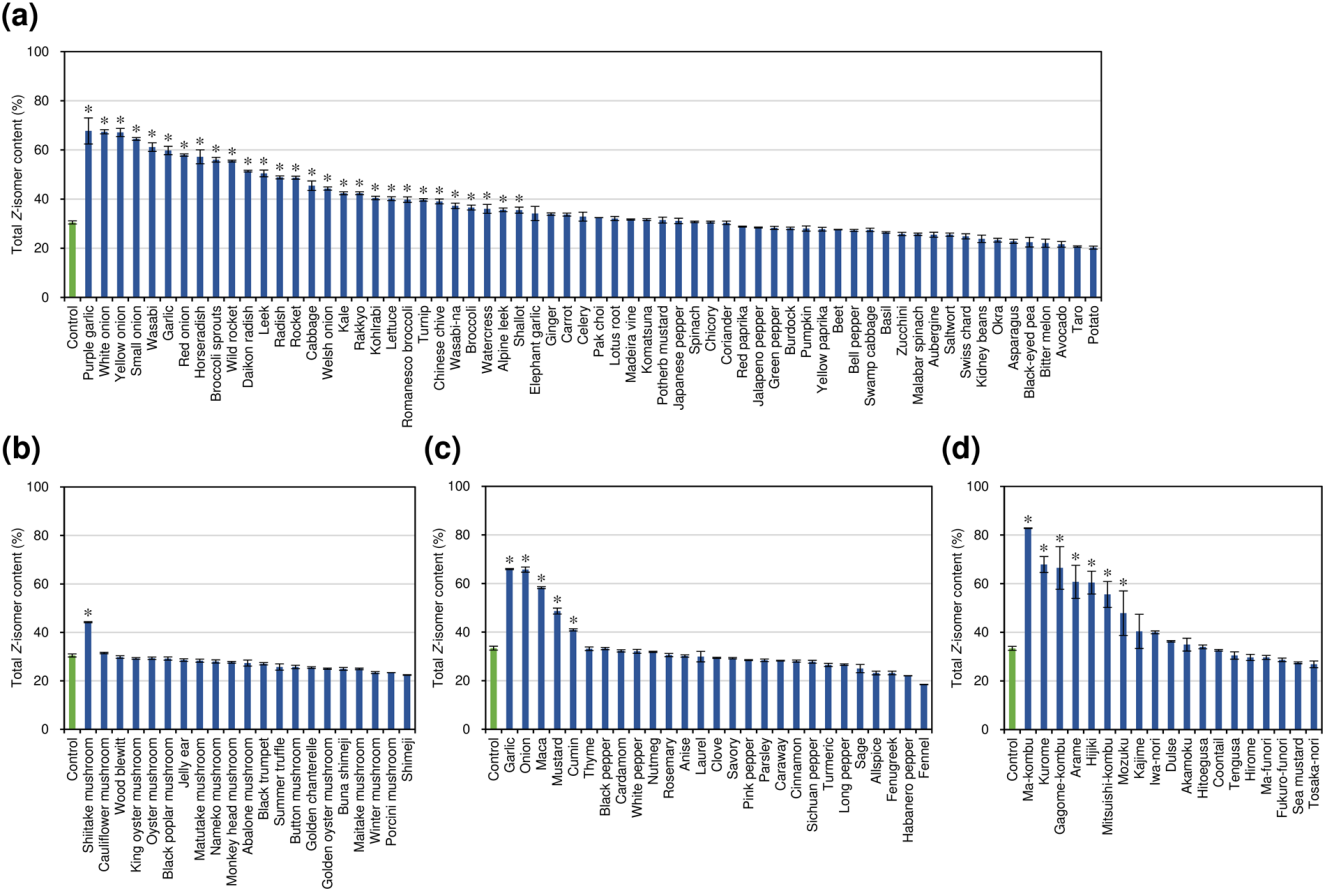
Effect of food ingredients on thermal *Z*-isomerization of lycopene contained in tomato puree: (**a**) effect of fresh vegetables; (**b)** effect of fresh mushrooms; (**c**) effect of dried spices and herbs; (**d**) effect of dried seaweeds. Error bars show standard deviation (*n* = 3). * indicates a statistically significant difference from the control group at *p* < 0.05 (*μ*_0_ < *μ*_i_). The concentration of lycopene before the heat treatment was (**a**,**b**) 0.13 mM or (**c**,**d**) 0.20 mM.

Here we report the effect of the addition of 131 food ingredients (fresh vegetables, fresh mushrooms, dried spices and herbs, and dried seaweeds) on the *Z*-isomer content of tomato puree (lycopene content, 12 mg/100 g; *Z*-isomer content, 9.2%) lycopene after heating at 80 °C for 1 h. Since the presence of mediators such as vegetable oils and organic solvents is necessary to promote thermal *Z*-isomerization of carotenoids^8,9^, a small amount of olive oil was added to the mixture. Typical chromatograms of the untreated and thermally treated samples are shown in Fig. 1, and total and each *Z*-isomer contents of the samples are summarized in Fig. 2 and Supplementary Tables S2–S5. In the case of adding fresh vegetables to the tomato puree (Fig. 2a), *Allium* sp. such as garlics (59.8–67.7%; Fig. 1d), onions (57.9–67.4%), and leek (50.4%), *Brassica* sp. such as cabbage (45.4%), *Raphanus* sp. such as radishes (48.8–51.4%), wasabi (*Wasabia japonica*) (61.1%), horseradish (*Armoracia rusticana*) (57.2%), wild rocket (*Diplotaxis tenuifolia*) (55.5%), and rocket (*Eruca sativa*) (48.7%) markedly promoted the *Z*-isomerization compared to the control (approximately 30%; Fig. 1b), in which distilled water was added in place of the food ingredients. When adding mushrooms, only shiitake mushroom (*Lentinus edodes*) (44.2%) significantly promoted the *Z*-isomerization (Figs 1c and 2b). With dried spices and herbs, garlic (65.9%), onion (65.7%), maca (*Lepidium meyenii*) (58.3%), and mustard (*B. juncea*) (48.6%) significantly enhanced the thermal *Z*-isomerization (Fig. 2c). Garlic and onion had a *Z*-isomerization promoting effect in both the fresh and dried states. de Alvarenga *et al*. (2017)^8^ reported that garlic had no noticeable effect on lycopene *Z*-isomerization by cooking. This is probably due to its low content in the formulations. Traditional tomato dishes such as *sofrito* and *gazpacho* as well as tomato sauce generally contain foods that have a *Z*-isomerization promoting effect such as onion, garlic, leek, cabbage and so on. Thus, these tomato dishes are not only tasty but also having high lycopene bioavailability. The addition of dried seaweeds, e.g., *Saccharina* sp. such as ma-kombu (*S. japonica*) (82.8%; Fig. 1e) and gagome-komb (*S. sculpera*) (66.5%), *Ecklonia* sp. such as kurome (*E. kurome*) (67.9%), and hijiki (*Sargassum fusiforme*) (60.4%) markedly promoted the *Z*-isomerization (Fig. 2d). In this heating condition (80 °C for 1 h), lycopene was scarcely decomposed in all tests using food ingredients, i.e., the remaining ratios of lycopene after the heat treatment were more than 90% (Supplementary Tables S2–S5).

Several studies indicated that (5*Z*)-lycopene exhibits greater bioavailability^10^ and antioxidant activity^11^ as well as storage stability among the *Z*-isomers^12^. Moreover, most recently, it was reported that (5*Z*)-lycopene have equal or higher antiobesity activity than the all-*E*-isomer^13^. Thus, to identify food ingredients that increase (5*Z*)-lycopene content is important. Of the 131 kinds of food ingredients investigated, the top five ingredients that promoted 5*Z*-isomerization of lycopene by heating were as follows: dried kurome (21.6%) >dried ma-kombu (20.9%) >fresh wild rocket (20.2%) >fresh rocket (20.1%) >fresh wasabi and dried hijiki (20.0%) (Supplementary Tables S2–S5).

To date, only onion and garlic have been reported as food ingredients that enhance thermal *Z*-isomerization of lycopene and no reports have compared their efficiencies among foods^7,8^. However, this study revealed many new food ingredients and clearly compared the *Z*-isomerization efficiencies among the foods. These findings are considered important to the proposal of processed tomato foods and supplement compositions that have high lycopene bioavailability. In fact, we analysed the *Z*-isomer content of commercially available raw tomatoes and tomato products such as tomato ketchup, pizza sauce, and tomato soup (Supplementary Fig. S1). Although the exact thermal history (sterilization condition), content of each food material, and lycopene content of the products are unknown, the products simultaneously containing oils and the foods ingredients that have the *Z*-isomerization promoting effect (garlic, onion, and kombu) showed high *Z*-isomer content.

In recent years, the low nutritional state of the elderly (caused by a reduction in appetite) has become a serious social problem and furthermore the carotenoid content, including lycopene, in the body decreases with aging, potentially playing a role in various diseases and sarcopenia^14,15^. Therefore, efforts to improve the absorbency of nutritional ingredients, such as the present research, are very important. Moreover, we report here for the first time that some seaweeds rapidly promote thermal *Z*-isomerization of (all-*E*)-lycopene. Seaweeds are rich in iodine^16^, and iodine deficiency remains a worldwide problem, with two billion individuals having insufficient iodine intake. This is also a serious problem in European countries, such as Italy and Portugal, where tomatoes are frequently eaten^17^. Based on the findings of this paper, it is therefore expected that new food menus using tomatoes and seaweeds will be proposed, alleviating this important global problem. Furthermore, tomato dishes containing high amounts of *Z*-isomers of lycopene have been traditionally eaten all over the world, and thus, it is considered that the *Z*-isomers are safe for human. In fact, we recently have confirmed the safety of tomato extract containing high amounts of *Z*-isomers of lycopene by bacterial reverse mutation assay and acute oral and 4-week repeated-dose oral toxicity tests using Wistar rats^18^.

### Causative components of enhancing lycopene *Z*-isomerization in foods

Using the above information of the food ingredients that promote thermal *Z*-isomerization of lycopene and our previous study^7^, we predicted some causative components. We recently reported that diallyl disulfide promoted thermal *Z*-isomerization of tomato lycopene, whereas diallyl sulfide and other sulfur compounds such as alliin and 2-furanmethanethiol which have no polysulfide bonds in the molecule did not^7^. Thus, components having polysulfide bonds could promote the thermal *Z*-isomerization of lycopene. In fact, *Allium* sp. such as onion, garlic and leek are rich in polysulfides, e.g., garlic contains around 2 mg/g wet weight of polysulfides such as allyl methyl disulfide and diallyl trisulfide^19^, while only shiitake mushroom contains polysulfides such as lenthionine (around 0.5 μmol/g dry weight)^20^ among the investigated mushrooms^20–23^. Moreover, several studies reported that electrophilic metals such as iron(III) chloride promote *Z*-isomerization of carotenoids^24^. Thus, electrophilic components contained in the foods could contribute to the promotion of thermal *Z*-isomerization. In this study, it was revealed that *Brassica* sp. and *Raphanus* sp. promoted *Z*-isomerization. These foods contain electrophilic isothiocyanates in considerable quantity: mustard seed (*B. juncea*) and daikon radish (*R. sativus*) contained 1.5 mmol/100 g wet weight and 108.8 μmol/g dry weight of isothiocyanates, respectively^25–27^. In addition, broccoli sprouts showed a higher *Z*-isomerization promoting effect than ripe broccoli. The reason is considered that broccoli sprouts contain a higher amount of isothiocyanates such as sulforaphane than ripe broccoli^28^. Further, wasabi and horseradish, which also promote *Z*-isomerization, contain a high amount of isothiocyanates^29^ and rocket and wild rocket contain carbon disulfide as an electrophilic component^30,31^. Food ingredients that did not promote the thermal *Z*-isomerization of tomato lycopene contain little or no polysulfides, isothiocyanates, and carbon disulfide, e.g., as for vegetables, herbs, and spices, ample studies have demonstrated that *Capsicum* sp., *Daucu*s sp., and *Piper* sp. contain little or no polysulfides, isothiocyanates, and carbon disulfide^32–35^. Moreover, seaweeds promoting the *Z*-isomerization reaction such as *Saccharin* sp. contain a large amount of iodine (around 2500 μg/g dry weight)^16^, whereas its content in seaweeds having almost no *Z*-isomerization promoting effect such as *Undaria* sp. is very low (around 50 μg/g dry weight)^16,36^. Iodine has traditionally been used as an *E*/*Z*-isomerization catalyst for double bonds^37^. Hence, we predicted that polysulfides, isothiocyanates, carbon disulfide, and iodine are the major causative compounds of the *Z*-isomerization and investigated the effect of their addition amount to tomato puree on the thermal *Z*-isomerization of (all-*E*)-lycopene.

**Figure 3:**
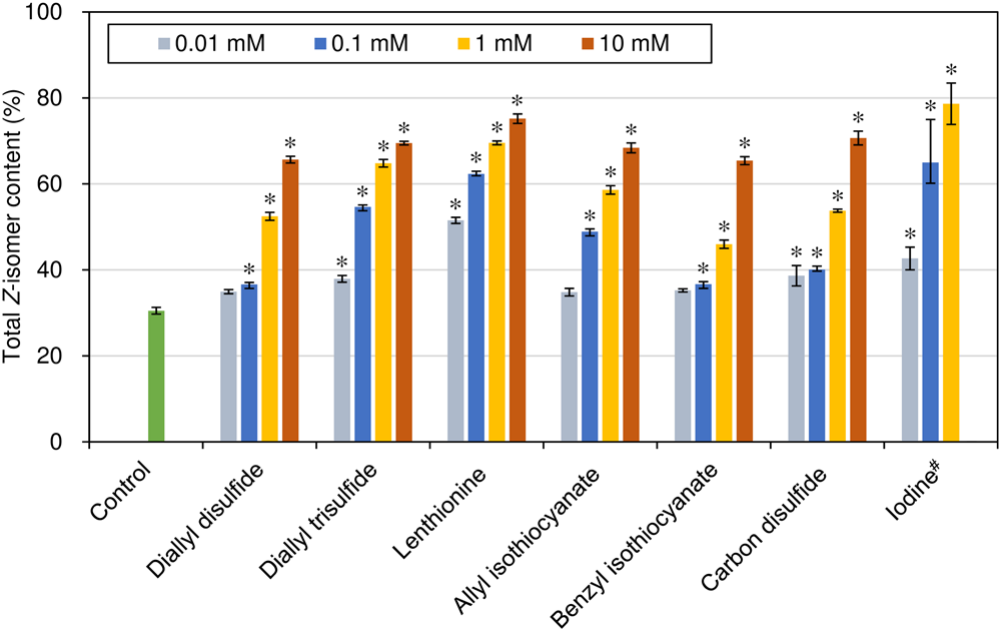
Effect of catalysts on thermal *Z*-isomerization of lycopene contained in tomato puree. Error bars show standard deviation (*n* = 3). ^#^At an iodine concentration of 10 mM, all lycopene was decomposed after the heat treatment; whereas, for all other catalysts and at all concentrations, the remaining ratios of total amount of lycopene isomers without decomposition after the treatment were more than 90% (Supplementary Table S6). *Indicates a statistically significant difference from the control group at *p* < 0.05 (*μ*_0_ < *μ*_i_). The concentration of lycopene before the heat treatment was 0.13 mM.

For all the above components, total *Z*-isomer contents of lycopene in tomato puree increased with the addition amount of the polysulfides, isothiocyanates, carbon disulfide, and iodine, and the remaining ratio of total amount of lycopene isomers without decomposition was more than 90%, other than in the case of iodine at the highest addition amount (10 mM; Fig. 3, Supplementary Table S6). Thus, it was revealed that polysulfides, isothiocyanates, carbon disulfide, and iodine widely contained in foods act as catalysts of *Z*-isomerization of (all-*E*)-lycopene and the appropriate amount added can efficiently promote *Z*-isomerization without lycopene decomposition. When comparing *Z*-isomerization efficiency among the catalysts at a fixed concentration, such as 1 mM, the efficiency was higher in the order of iodine (78.5 ± 4.8%) >lenthionine (69.5 ± 0.4%) >diallyl trisulfide (64.8 ± 0.9%) >allyl isothiocyanate (58.6 ± 1.0%) >carbon disulfide (53.8 ± 0.4%) >diallyl disulfide (52.5 ± 0.9%) >benzyl isothiocyanate (46.0 ± 1.0%) (Fig. 3). Kombus (*Saccharin sp*.) contains an especially high amount of iodine among seaweeds^16,36^, and in fact, iodine showed the highest isomerization efficiency of the catalysts. As for polysulfides, an increase in the number of disulfide bonds increased the thermal *Z*-isomerization efficiency.

The discovery of food-derived catalysts could facilitate innovative changes to carotenoid processing (Supplementary Fig. S2). Generally, (all-*E*)-carotenoids are characterized by high hydrophobicity and crystallinity; thus, they are insoluble in water and sparingly soluble in oils and polar solvents^38,39^. These properties result in a decrease in the processing efficiencies of carotenoids, namely in extraction, micronization, and emulsification. However, *Z*-isomerization of carotenoids induces changes in physicochemical properties such as solubility and crystallinity, e.g., the solubility in ethanol of astaxanthin *Z*-isomers was more than 250 times higher than that of the all-*E*-isomer^38^. Very recently, several studies reported that *Z*-isomerization treatment of carotenoids prior to extraction^39^, micronization^40^, and emulsification^41^ drastically improved those processing efficiencies. Hence, by adding *Z*-isomerization catalysts to the processing of carotenoids, the processing efficiency could be simultaneously improved with *Z*-isomerization. Moreover, since polysulfides, isothiocyanates, and carbon disulfide exhibit relatively low volatility, they could be removed under reduced pressure and heating conditions: solvent removal process in carotenoids processing (Supplementary Fig. S2). The food-derived catalysts found in this study would catalyze food components containing double bonds other than carotenoids and further progress in this research area is expected in the future.

## Materials and Methods

### Materials

Analytical-grade acetone was purchased from Sigma-Aldrich Co., Ltd. (Poole, Dorset, UK) and HPLC-grade hexane was obtained from Kanto Chemical Co., Inc. (Tokyo, Japan). Diallyl disulfide, allyl isothiocyanate, iodine, and *N*,*N*-diisopropylethylamine (DIPEA) were obtained from Tokyo Chemical Industry Co., Ltd. (Tokyo, Japan). Benzyl isothiocyanate and carbon disulfide were purchased Fujifilm Wako Pure Chemical Corp. (Osaka, Japan). Diallyl trisulfide and lenthionine were obtained from LKT Laboratories, Inc. (St. Paul, MN, USA) and Combi-Blocks, Inc. (San Diego, CA, USA), respectively. (all-*E*)-Lycopene (normal-phase HPLC, ≥98.0% purity) was purified from tomato oleoresin (Lyc-O-Mato^®^ 15%, LycoRed Ltd., Beer-Sheva, Israel) according to the previous method^9^. Heat sterilized tomato puree (lycopene content, 12 mg/100 g, doubling dilution; total *Z***-**isomer content, 9.2%) was purchased from Kagome Co., Ltd. (Tokyo, Japan) and pure olive oil was purchased from Nissin Oillio Group, Ltd. (Tokyo, Japan). The other food ingredients, i.e., fresh vegetables, fresh mushrooms, dried spices and herbs, and dried seaweeds, were purchased at a local supermarket in Nagoya, Japan. The fresh vegetables and mushrooms were not sterilized and the moisture content would over approximately 50%^42,43^, and before used, fresh vegetables were washed the surface with water but the mushrooms were not washed. Dried spices, herbs, and seaweeds were not sterilized but some of them were sterilized such as cumin, nutmeg, turmeric, and hijiki. The moisture contents of the dried food ingredients were less than approximately 15%, which were determined by heat drying type moisture analyzer (ML-50; A&D Co., Ltd., Tokyo, Japan) or were acquired from the suppliers.

### Thermal *Z*-isomerization of (all-*E*)-lycopene contained in tomato puree

When using fresh food ingredients, the mixing ratio of tomato puree, fresh foods, and olive oil was adjusted to 60:35:5 (w/w/w), whereas when using dried foods, the ratio was adjusted to 90:5:5 (w/w/w). As controls of the above tests, the heating tests of the mixture in which distilled water was added in place of the food ingredients, i.e., the tomato puree, water, and olive oil was adjusted to 60:35:5 (w/w/w) or 90:5:5 (w/w/w), were carried out. Olive oil was added to the mixture to promote thermal *Z*-isomerization of lycopene^9^. Each mixture was homogenized using a food processor (SJM-180G; Siroca, Inc., Tokyo, Japan) for 1 min and approximately 20 g of each sample was transferred to a 100-mL screw-capped glass bottle. Then, the headspace was purged with nitrogen gas, and immediately, the bottle was tightly capped to minimize oxygen exposure. The mixtures were heated in a water bath at 80 °C for 1 h in the dark to isomerize (all-*E*)-lycopene to the *Z*-isomers^44,45^. Similarly, when investigating the effect of the food-derived catalysts (polysulfides, isothiocyanates, carbon disulfide, and iodine) on *Z*-isomerization, they were added to the mixture of tomato puree, i.e., 60:35:5 (w/w/w) at a mixing ratio of tomato puree, distilled water, and olive oil, to a final concentration of 0.01, 0.1, 1, and 10 mM, and then the thermal *Z*-isomerization was conducted using the same condition as above. This added amount of sulfur compounds was based on the amounts contained in the food ingredients^16,19,20,27,31^.

### Extraction of lycopene isomers

The extraction of lycopene isomers from the samples was carried out using acetone, according to a previously described method^9,46^. Unless specifically mentioned, all procedures were conducted at room temperature, and light exposure was kept to a minimum throughout the extraction. One gram of samples was weighed into a 30-mL screw-capped glass bottle and 30 mL of acetone was added. Lycopene isomers contained in the samples were extracted by ultrasonic treatment for 10 min, and then the residue was removed by vacuum filtration using number 2 filter paper (Advantec Co., Ltd., Tokyo, Japan). If any colour remained in the residue, it was rinsed with acetone until the residue was colourless. The extract solution containing lycopene isomers was evaporated to dryness under reduced pressure at 40 °C, dissolved in 10 mL of hexane, and then filtered through a 0.2-μm polytetrafluoroethylene membrane filter (Advantec Co., Ltd.) for normal-phase HPLC analysis. The above method was also applied to the commercially available raw tomatoes and tomato processed foods such as tomato juice, tomato puree, tomato ketchup, pizza sauce, and tomato soup.

### HPLC analysis

Normal-phase HPLC analysis of lycopene isomers was conducted according to previously described conditions^9,47^. Briefly, the mobile phase consisted of hexane containing 0.075% *N*,*N*-diisopropylethylamine and lycopene isomers were separated by three Nucleosil 300–5 columns connected in tandem (3 × 250 mm length, 4.6 mm inner diameter, 5 μm particle size; GL Sciences Inc., Tokyo, Japan). The isocratic flow rate and column temperature were set at 1.0 mL/min and 35 °C, respectively. The quantification of lycopene isomers was performed by peak area integration at 460 nm, at which the differences in molar extraction coefficients among lycopene isomers are relatively small^47–49^. The peaks of lycopene isomers such as 5*Z*-, 9*Z*-, and 13*Z*-isomers were identified using the HPLC retention times, UV spectra, and relative intensities of the *Z*-peak at approximately 360 nm to the absorption maximum of the isomer (% *D*_B_/D_II_) (Supplementary Table S1), as described previously^9,47–49^. The total (or each) lycopene *Z*-isomer content (%) was estimated by HPLC peak area at 460 nm as the amount of total *Z*-isomers (or each *Z*-isomer) to the amount of total lycopene isomers including the all-*E*-isomer. The remaining ratio of lycopene (%) after the heat treatment was determined by comparing HPLC peak area of total lycopene isomers before and after the treatment.

### Statistical analysis

All data were collected in triplicate and are expressed as the mean ± standard deviation. For the studies of the effect of food ingredients and catalysts on the thermal *Z*-isomerization of lycopene, Dunnett’s test (*p* < 0.05) was used for evaluating statistical significance compared to the control group using JMP software (version 14.1.0; SAS Institute Inc., Cary, NC, USA).

## Supplementary information

Table S1, Table S2, Table S3, Table S4, Table S5, Table S6, Figure S1, Figure S2
