# Supplementary material for: Enhanced Z-isomerization of tomato lycopene through the optimal combination of food ingredients
Source: Sci Rep. 2019 May 28;9:7979. doi: 10.1038/s41598-019-44177-4 (PMC6538647; doi:10.1038/s41598-019-44177-4)

***Supplementary Material***

**Enhanced *Z*-isomerization of tomato lycopene through the optimal combination of food ingredients**

Masaki Honda^1^, Hakuto Kageyama^1^, Takashi Hibino^1^, Ryota Takemura^2^, Motonobu Goto^3^, Tetsuya Fukaya^2,4^

^1^ Faculty of Science & Technology, Meijo University, Shiogamaguchi, Tempaku-ku, Nagoya 468-8502, Japan

^2^ Innovation Division, Kagome Company, Limited, Nishitomiyama, Nasushiobara 329-2762, Japan

^3^ Department of Materials Process Engineering, Nagoya University, Furo-cho, Chikusa-ku, Nagoya 464-8603, Japan^4^ Institutes of Innovation for Future Society, Nagoya University, Furo-cho, Chikusa-ku, Nagoya 464-8603, Japan

^4^ Institutes of Innovation for Future Society, Nagoya University, Furo-cho, Chikusa-ku, Nagoya 464-8603, Japan

Correspondence and requests for materials should be addressed to M.H. (honda@meijo-u.ac.jp) and T.F. (Tetsuya_Fukaya@kagome.co.jp).

**Table S1. Absorption maxima (*λ*_max_) and relative intensities of the *Z*-peaks (%*D*_B_/*D*_II_) for geometrical lycopene isomers separated and observed using normal-phase HPLC**^a^

|  |  | *λ*_max_ (nm) | |  | % *D*_B_/*D*_II_ | |
| --- | --- | --- | --- | --- | --- | --- |
| Peak | Lycopene isomer^b^ | In-line | Reported^b^ |  | In-line | Reported^b^ |
| 1 | (9*Z*,13′*Z*) | 360, 433, 458, 489 | 361, 434, 459, 489 |  | 28.6 | 31 |
| 2 | u.z. | 360, 433, 458, 489 | – |  | 26.6 | – |
| 3 | (9*Z*, 9′*Z*) | 360, 433, 458, 489 | 361, 435, 460, 491 |  | 14.5 | 11 |
| 4 | u.z. | 360, 438, 464, 496 | – |  | 41.1 | – |
| 5 | u.z. | 360, 438, 465, 496 | – |  | 43.1 | – |
| 6 | u.z. | 360, 433, 458, 489 | – |  | 26.9 | – |
| 7 | (5*Z*,13′*Z*) | 360, 440, 465, 496 | 361, 439, 465, 497 |  | 43.4 | 56 |
|  | (13*Z*) | 360, 439, 464, 495 | 361, 437, 463, 494 |  | 43.9 | 59 |
| 8 | u.z. | 360, 433, 459, 489 | – |  | 33.4 | – |
| 9 | u.z. | 360, 433, 458, 489 | – |  | 12.6 | – |
| 10 | u.z. | 360, 433, 459, 489 | – |  | 26.7 | – |
| 11 | (5*Z*,9′*Z*) | 360, 438, 464, 496 | 361, 442, 467, 497 |  | 13.4 | 13 |
| 12 | u.z. | 360, 433, 458, 489 | – |  | 21.7 | – |
| 13 | u.z. | 360, 437, 463, 493 | – |  | 15.0 | – |
|  | (9*Z*) | 360, 438, 464, 495 | 361, 442, 467, 497 |  | 14.0 | 13 |
| 14 | (5*Z*,9*Z*,5′*Z*) | 360, 438, 464, 496 | 361, 442, 467, 497 |  | 13.5 | 13 |
| 15 | (5*Z*,9*Z*) | 360, 438, 464, 496 | 361, 440, 460, 497 |  | 13.7 | 13 |
| 16 | (5*Z*,5′*Z*) | 444, 470, 501 | 443, 470, 501 |  | n.d. | n.d. |
|  | (all-*E*) | 444, 470, 501 | 444, 471, 502 |  | n.d. | n.d. |
|  | (5*Z*) | 444, 470, 501 | 444, 471, 502 |  | n.d. | n.d. |

^a^Values and peak designations were obtained from the chromatograms in Figure 1. u.z., unidentified *Z*-isomer of lycopene. –, not assigned. n.d., not detected substantially.

^b^Tentatively assigned in the literatures^9,47–49^.

**Table S2. Effect of various fresh vegetables on the thermal *E*/*Z*-isomerization of lycopene contained in tomato puree**

| Scientific name | General name | Remaining lycopene^a^ | Content (%)^b^ | | | | | |
| --- | --- | --- | --- | --- | --- | --- | --- | --- |
|  |  |  | (All-*E*) | Total *Z*^c^ | (5*Z*) | (9*Z*) | (13*Z*) | Other *Z*^d^ |
| (Control) | – | 96.6 ± 2.7 | 69.5 ± 0.6 | 30.5 ± 0.6 | 3.0 ± 0.2 | 3.5 ± 0.2 | 16.6 ± 0.1 | 7.4 ± 0.4 |
| *Abelmoschus esculentus* | Okra | 99.7 ± 0.2 | 76.7 ± 0.8^e^ | 23.3 ± 0.8^e^ | 4.2 ± 0.0 | 8.3 ± 0.4^e^ | 7.9 ± 0.4^e^ | 2.8 ± 0.4^e^ |
| *Allium ampeloprasum* | Elephant garlic | 98.6 ± 0.9 | 65.9 ± 2.9 | 34.1 ± 2.9 | 7.8 ± 0.4^e^ | 5.8 ± 0.5^e^ | 7.3 ± 1.1^e^ | 13.3 ± 0.8^e^ |
| *Allium ampeloprasum* | Leek | 98.1 ± 1.4 | 49.6 ± 1.4^e^ | 50.4 ± 1.4^e^ | 12.3 ± 0.2^e^ | 8.7 ± 0.2^e^ | 9.0 ± 0.4^e^ | 20.4 ± 0.8^e^ |
| *Allium ascalonicum* | Shallot | 91.0 ± 2.3 | 64.5 ± 1.2^e^ | 35.5 ± 1.2^e^ | 9.0 ± 0.1^e^ | 6.0 ± 0.3^e^ | 6.9 ± 0.4^e^ | 13.6 ± 1.3^e^ |
| *Allium cepa* | White onion | 96.8 ± 0.8 | 32.6 ± 0.8^e^ | 67.4 ± 0.8^e^ | 15.7 ± 0.2^e^ | 12.0 ± 0.7^e^ | 11.8 ± 2.7^e^ | 27.8 ± 2.3^e^ |
| *Allium cepa* | Yellow onion | 95.3 ± 1.5 | 32.9 ± 1.7^e^ | 67.1 ± 1.7^e^ | 15.5 ± 0.4^e^ | 9.4 ± 0.5^e^ | 12.4 ± 0.5^e^ | 29.8 ± 0.8^e^ |
| *Allium cepa* | Red onion | 97.4 ± 0.8 | 42.1 ± 0.5^e^ | 57.9 ± 0.5^e^ | 13.7 ± 0.1^e^ | 7.0 ± 0.5^e^ | 9.9 ± 0.4^e^ | 27.3 ± 0.8^e^ |
| *Allium cepa* | Small onion | 95.6 ± 2.7 | 35.5 ± 0.6^e^ | 64.5 ± 0.6^e^ | 14.9 ± 0.2^e^ | 8.9 ± 0.1^e^ | 7.2 ± 0.6^e^ | 33.5 ± 0.3^e^ |
| *Allium chinense* | Rakkyo | 96.8 ± 1.2 | 57.6 ± 0.6^e^ | 42.4 ± 0.6^e^ | 9.9 ± 0.1^e^ | 7.5 ± 0.4^e^ | 7.3 ± 0.2^e^ | 17.8 ± 0.3^e^ |
| *Allium fistulosum* | Welsh onion | 98.4 ± 0.9 | 55.7 ± 0.6^e^ | 44.3 ± 0.6^e^ | 14.4 ± 0.1^e^ | 9.0 ± 0.4^e^ | 7.5 ± 0.6^e^ | 13.4 ± 0.5^e^ |
| *Allium sativum* | Garlic | 94.2 ± 1.3 | 40.2 ± 1.7^e^ | 59.8 ± 1.7^e^ | 14.8 ± 0.7^e^ | 8.4 ± 0.7^e^ | 11.0 ± 0.5^e^ | 25.6 ± 1.1^e^ |
| *Allium sativum* | Purple garlic | 95.8 ± 1.6 | 32.3 ± 5.3^e^ | 67.7 ± 5.3^e^ | 16.4 ± 0.9^e^ | 11.0 ± 0.7^e^ | 7.0 ± 0.8^e^ | 33.3 ± 3.0^e^ |
| *Allium tuberosum* | Chinese chive | 96.4 ± 1.1 | 61.0 ± 1.0^e^ | 39.0 ± 1.0^e^ | 14.7 ± 0.4^e^ | 10.6 ± 0.2^e^ | 7.7 ± 0.7^e^ | 6.1 ± 0.6 |
| *Allium victorialis* | Alpine leek | 96.7 ± 3.3 | 64.4 ± 0.8^e^ | 35.6 ± 0.8^e^ | 15.1 ± 0.7^e^ | 10.9 ± 0.5^e^ | 4.9 ± 0.2^e^ | 4.7 ± 0.1 |
| *Anredera cordifolia* | Madeira vine | 98.1 ± 1.6 | 68.3 ± 0.2 | 31.7 ± 0.2 | 5.2 ± 0.4^e^ | 11.0 ± 0.2^e^ | 9.8 ± 0.3^e^ | 5.7 ± 0.3 |
| *Arctium lappa* | Burdock | 96.3 ± 1.2 | 71.9 ± 0.5 | 28.1 ± 0.5 | 3.9 ± 0.0 | 3.9 ± 0.1 | 12.1 ± 0.2^e^ | 8.1 ± 0.5 |
| *Armoracia rusticana* | Horseradish | 96.9 ± 0.2 | 42.8 ± 2.8^e^ | 57.2 ± 2.8^e^ | 12.7 ± 0.4^e^ | 8.5 ± 0.1^e^ | 8.2 ± 0.7^e^ | 27.9 ± 2.1^e^ |
| *Asparagus officinalis* | Asparagus | 93.1 ± 0.5 | 77.2 ± 0.8^e^ | 22.8 ± 0.8^e^ | 4.4 ± 0.3^e^ | 9.9 ± 0.6^e^ | 5.5 ± 0.3^e^ | 2.9 ± 0.7^e^ |
| *Apium graveolens* | Celery | 98.9 ± 3.6 | 67.1 ± 1.8 | 32.9 ± 1.8 | 9.2 ± 1.0^e^ | 9.9 ± 0.1^e^ | 7.0 ± 1.5^e^ | 6.7 ± 1.3 |
| *Basella alba* | Malabar spinach | 97.9 ± 1.8 | 74.3 ± 0.5^e^ | 25.7 ± 0.5^e^ | 5.4 ± 0.2^e^ | 11.4 ± 0.2^e^ | 5.3 ± 0.2^e^ | 3.6 ± 0.3^e^ |
| *Beta vulgaris* | Beet | 93.3 ± 1.2 | 72.4 ± 0.1 | 27.6 ± 0.1 | 3.8 ± 0.1 | 3.8 ± 0.1 | 12.2 ± 0.2^e^ | 7.7 ± 0.0 |
| *Beta vulgaris* | Swiss chard | 94.8 ± 0.8 | 75.2 ± 1.0^e^ | 24.8 ± 1.0^e^ | 4.8 ± 0.8^e^ | 10.9 ± 0.6^e^ | 4.9 ± 0.7^e^ | 4.3 ± 0.4^e^ |
| *Brassica juncea* | Potherb mustard | 97.4 ± 2.0 | 68.6 ± 1.2 | 31.4 ± 1.2 | 9.0 ± 0.1^e^ | 11.5 ± 0.3^e^ | 5.5 ± 0.5^e^ | 5.5 ± 0.5 |
| *Brassica juncea* | Wasabi-na | 95.8 ± 1.0 | 62.7 ± 1.1^e^ | 37.3 ± 1.1^e^ | 13.9 ± 0.3^e^ | 11.3 ± 0.6^e^ | 4.6 ± 0.5^e^ | 7.5 ± 0.4 |
| *Brassica oleracea* | Broccoli sprouts | 96.2 ± 2.1 | 44.0 ± 0.9^e^ | 56.0 ± 0.9^e^ | 19.8 ± 0.4^e^ | 11.2 ± 0.2^e^ | 9.5 ± 0.7^e^ | 15.5 ± 0.9^e^ |
| *Brassica oleracea* | Broccoli | 99.0 ± 1.0 | 63.4 ± 1.0^e^ | 36.6 ± 1.0^e^ | 15.2 ± 1.0^e^ | 9.6 ± 0.4^e^ | 4.5 ± 0.4^e^ | 7.2 ± 1.5 |
| *Brassica oleracea* | Romanesco broccoli | 96.9 ± 2.4 | 60.2 ± 1.1^e^ | 39.8 ± 1.1^e^ | 9.8 ± 0.2^e^ | 9.3 ± 0.1^e^ | 8.0 ± 1.1^e^ | 12.6 ± 0.3^e^ |
| *Brassica oleracea* | Cabbage | 96.7 ± 1.8 | 54.6 ± 1.9^e^ | 45.4 ± 1.9^e^ | 16.5 ± 0.5^e^ | 9.3 ± 0.4^e^ | 6.4 ± 0.7^e^ | 13.3 ± 1.3^e^ |
| *Brassica oleracea* | Kale | 93.2 ± 2.7 | 57.6 ± 0.6^e^ | 42.4 ± 0.6^e^ | 17.4 ± 0.5^e^ | 11.0 ± 0.3^e^ | 3.7 ± 0.2^e^ | 10.3 ± 0.7^e^ |
| *Brassica oleracea* | Kohlrabi | 92.8 ± 3.5 | 59.6 ± 0.7^e^ | 40.4 ± 0.7^e^ | 9.7 ± 0.4^e^ | 8.8 ± 0.0^e^ | 7.0 ± 0.3^e^ | 14.9 ± 0.9^e^ |
| *Brassica rapa* | Turnip | 97.5 ± 0.4 | 60.3 ± 0.5^e^ | 39.7 ± 0.5^e^ | 8.4 ± 0.3^e^ | 7.6 ± 0.2^e^ | 10.7 ± 0.1^e^ | 13.1 ± 0.5^e^ |
| *Brassica rapa* | Pak choi | 93.8 ± 2.1 | 67.5 ± 0.0 | 32.5 ± 0.0 | 8.5 ± 0.1^e^ | 11.3 ± 0.1^e^ | 5.3 ± 0.4^e^ | 7.3 ± 0.5 |
| *Brassica rapa* | Komatsuna | 94.8 ± 2.5 | 68.4 ± 0.4 | 31.6 ± 0.4 | 9.7 ± 0.2^e^ | 12.2 ± 0.2^e^ | 4.4 ± 0.1^e^ | 5.3 ± 0.3 |
| *Capsicum annuum* | Green pepper | 94.2 ± 5.4 | 71.7 ± 0.6 | 28.3 ± 0.6 | 7.3 ± 0.5^e^ | 9.9 ± 0.5^e^ | 6.1 ± 0.3^e^ | 5.0 ± 0.3 |
| *Capsicum annuum* | Jalapeno pepper | 92.0 ± 1.7 | 71.6 ± 0.2 | 28.4 ± 0.2 | 6.9 ± 0.0^e^ | 11.1 ± 0.1^e^ | 6.5 ± 0.2^e^ | 4.0 ± 0.1^e^ |
| *Capsicum annuum* | Bell pepper | 92.1 ± 1.7 | 72.8 ± 0.4 | 27.2 ± 0.4 | 7.1 ± 0.1^e^ | 10.0 ± 0.2^e^ | 5.2 ± 0.3^e^ | 4.9 ± 0.1 |
| *Capsicum annuum* | Red paprika | 97.1 ± 1.0 | 71.2 ± 0.2 | 28.8 ± 0.2 | 3.9 ± 0.1 | 3.9 ± 0.1 | 12.3 ± 0.6^e^ | 8.8 ± 0.3 |
| *Capsicum annuum* | Yellow paprika | 95.7 ± 2.2 | 72.2 ± 0.8 | 27.8 ± 0.8 | 3.3 ± 0.4 | 2.8 ± 0.1 | 13.1 ± 0.2^e^ | 8.5 ± 0.4 |
| *Cichorium intybus* | Chicory | 96.9 ± 0.3 | 69.4 ± 0.4 | 30.6 ± 0.4 | 7.0 ± 0.1^e^ | 7.8 ± 0.3^e^ | 9.2 ± 0.3^e^ | 6.6 ± 0.2 |
| *Colocasia esculenta* | Taro | 96.2 ± 1.0 | 79.3 ± 0.3^e^ | 20.7 ± 0.3^e^ | 3.5 ± 0.1 | 2.7 ± 0.2 | 8.6 ± 0.3^e^ | 5.9 ± 0.3 |
| *Coriandrum sativum* | Coriander | 97.4 ± 2.5 | 69.7 ± 0.7 | 30.3 ± 0.7 | 7.2 ± 0.1^e^ | 12.4 ± 0.7^e^ | 6.5 ± 0.4^e^ | 4.3 ± 0.2^e^ |
| *Cucurbita maxima* | Pumpkin | 97.1 ± 0.9 | 72.0 ± 1.0 | 28.0 ± 1.0 | 3.6 ± 0.4 | 3.3 ± 0.1 | 15.0 ± 1.2 | 6.1 ± 1.6 |
| *Cucurbita pepo* | Zucchini | 93.6 ± 2.4 | 74.2 ± 0.7^e^ | 25.8 ± 0.7^e^ | 5.1 ± 0.4^e^ | 8.5 ± 0.3^e^ | 8.0 ± 0.3^e^ | 4.2 ± 0.3^e^ |
| *Daucus carota* | Carrot | 99.8 ± 1.4 | 66.3 ± 0.6 | 33.7 ± 0.6 | 4.7 ± 0.1^e^ | 5.0 ± 0.0^e^ | 15.0 ± 0.2 | 8.9 ± 0.6 |
| *Diplotaxis tenuifolia* | Wild rocket | 96.5 ± 2.6 | 44.5 ± 0.4^e^ | 55.5 ± 0.4^e^ | 20.2 ± 0.8^e^ | 7.7 ± 0.4^e^ | 7.4 ± 0.2^e^ | 20.1 ± 0.3^e^ |
| *Eutrema japonicum* | Wasabi | 99.4 ± 0.5 | 38.9 ± 1.7^e^ | 61.1 ± 1.7^e^ | 20.0 ± 0.4^e^ | 12.6 ± 0.2^e^ | 6.0 ± 1.0^e^ | 22.5 ± 0.9^e^ |
| *Eruca sativa* | Rocket | 99.3 ± 0.2 | 51.3 ± 0.6^e^ | 48.7 ± 0.6^e^ | 20.1 ± 0.2^e^ | 9.6 ± 0.2^e^ | 9.2 ± 0.5^e^ | 9.8 ± 0.1 |
| *Lactuca sativa* | Lettuce | 92.0 ± 1.5 | 59.8 ± 0.8^e^ | 40.2 ± 0.8^e^ | 6.9 ± 0.1^e^ | 8.1 ± 0.3^e^ | 12.6 ± 0.2^e^ | 12.5 ± 0.9^e^ |
| *Lpomoea aquatica* | Swamp cabbage | 95.8 ± 1.5 | 72.5 ± 0.7 | 27.5 ± 0.7 | 7.0 ± 0.1^e^ | 11.3 ± 0.3^e^ | 4.5 ± 0.1^e^ | 4.8 ± 0.4 |
| *Momordica charantia* | Bitter melon | 95.8 ± 1.3 | 78.0 ± 1.7^e^ | 22.0 ± 1.7^e^ | 4.5 ± 0.0^e^ | 8.9 ± 0.8^e^ | 5.4 ± 0.5^e^ | 3.3 ± 0.3^e^ |
| *Nasturtium officinale* | Watercress | 93.5 ± 1.3 | 64.0 ± 1.8^e^ | 36.0 ± 1.8^e^ | 14.4 ± 0.6^e^ | 10.7 ± 0.2^e^ | 4.5 ± 0.6^e^ | 6.4 ± 0.9 |
| *Nelumbo nucifera* | Lotus root | 98.4 ± 1.0 | 67.9 ± 0.8 | 32.1 ± 0.8 | 3.6 ± 0.2 | 4.1 ± 0.1 | 15.0 ± 0.5 | 9.4 ± 0.5 |
| *Ocimum basilicum* | Basil | 97.1 ± 1.2 | 73.5 ± 0.3 | 26.5 ± 0.3 | 4.9 ± 0.3^e^ | 12.8 ± 0.1^e^ | 5.0 ± 0.3^e^ | 3.8 ± 0.4^e^ |
| *Persea americana* | Avocado | 95.4 ± 3.9 | 78.4 ± 1.2^e^ | 21.6 ± 1.2^e^ | 4.0 ± 0.1 | 6.4 ± 0.6^e^ | 7.1 ± 0.5^e^ | 4.1 ± 0.5^e^ |
| *Phaseolus vulgaris* | Kidney bean | 96.3 ± 1.6 | 76.2 ± 1.5^e^ | 23.8 ± 1.5^e^ | 3.7 ± 0.2 | 12.0 ± 0.3^e^ | 4.9 ± 0.5^e^ | 3.2 ± 0.9^e^ |
| *Raphanus sativus* | Daikon radish | 92.8 ± 1.7 | 48.6 ± 0.4^e^ | 51.4 ± 0.4^e^ | 10.6 ± 0.4^e^ | 7.0 ± 2.0^e^ | 9.8 ± 0.1^e^ | 24.0 ± 2.2^e^ |
| *Raphanus sativus* | Radish | 95.6 ± 5.1 | 51.2 ± 0.6^e^ | 48.8 ± 0.6^e^ | 10.7 ± 0.2^e^ | 8.8 ± 0.0^e^ | 8.6 ± 0.3^e^ | 20.8 ± 1.0^e^ |
| *Salsola komarovii* | Saltwort | 96.7 ± 0.9 | 74.5 ± 0.7^e^ | 25.5 ± 0.7^e^ | 5.1 ± 0.2^e^ | 12.0 ± 0.4^e^ | 4.9 ± 0.2^e^ | 3.5 ± 0.3^e^ |
| *Solanum melongena* | Aubergine | 95.3 ± 1.5 | 74.5 ± 1.0^e^ | 25.5 ± 1.0^e^ | 4.0 ± 0.1 | 4.1 ± 0.2 | 10.2 ± 0.3^e^ | 7.3 ± 0.4 |
| *Solanum tuberosum* | Potato | 98.2 ± 1.3 | 79.7 ± 0.6^e^ | 20.3 ± 0.6^e^ | 2.6 ± 0.2 | 1.6 ± 0.1^e^ | 11.0 ± 0.3^e^ | 5.0 ± 0.4 |
| *Spinacia oleracea* | Spinach | 97.1 ± 1.0 | 69.3 ± 0.3 | 30.7 ± 0.3 | 5.5 ± 0.0^e^ | 11.8 ± 0.1^e^ | 7.7 ± 0.2^e^ | 5.7 ± 0.2 |
| *Vigna unguiculata* | Black‐eyed pea | 95.1 ± 1.4 | 77.5 ± 2.0^e^ | 22.5 ± 2.0^e^ | 3.8 ± 0.3 | 10.6 ± 0.9^e^ | 5.4 ± 1.2^e^ | 2.7 ± 0.6^e^ |
| *Zanthoxylum piperitum* | Japanese pepper | 98.8 ± 0.2 | 68.9 ± 1.1 | 31.1 ± 1.1 | 5.4 ± 0.4^e^ | 10.1 ± 0.7^e^ | 11.3 ± 0.5^e^ | 4.3 ± 0.2^e^ |
| *Zingiber officinale* | Ginger | 99.0 ± 1.1 | 66.1 ± 0.5 | 33.9 ± 0.5 | 3.8 ± 0.0 | 2.4 ± 0.2 | 24.9 ± 1.2^e^ | 2.8 ± 0.5^e^ |

The thermal treatment of the mixture of tomato puree, fresh vegetable, and olive oil (60:35:5, w/w) was carried out at 80 °C for 1 h.

^a^Remaining ratio of total amount of lycopene isomers without decomposition by the heat treatment.

^b^Percentage content of *Z*-isomers of lycopene relative to the total amount of lycopene.

^c^Total content of *Z*-isomers of lycopene.

^d^Sum of *Z*-isomers of lycopene other than 5*Z*-, 9*Z*-, and 13*Z*-forms.

^e^Significant, *p* < 0.05 versus control (*μ*_0_ ≠ *μ*_i_).

**Table S3. Effect of various fresh mushrooms on the thermal *E*/*Z*-isomerization of lycopene contained in tomato puree**

| Scientific name | General name | Remaining lycopene^a^ | Content (%)^b^ | | | | | |
| --- | --- | --- | --- | --- | --- | --- | --- | --- |
|  |  |  | (All-*E*) | Total *Z*^c^ | (5*Z*) | (9*Z*) | (13*Z*) | Other *Z*^d^ |
| (Control) | – | 96.6 ± 2.7 | 69.5 ± 0.6 | 30.5 ± 0.6 | 3.0 ± 0.2 | 3.5 ± 0.2 | 16.6 ± 0.1 | 7.4 ± 0.4 |
| *Agaricus bisporus* | Button mushroom | 97.4 ± 2.6 | 74.3 ± 0.7 | 25.7 ± 0.7 | 3.3 ± 0.2 | 3.0 ± 0.4 | 12.6 ± 0.2^e^ | 6.8 ± 0.4 |
| *Agrocybe cylindracea* | Black Poplar Mushroom | 96.6 ± 2.4 | 70.8 ± 0.7 | 29.2 ± 0.7 | 3.1 ± 0.1 | 3.9 ± 0.2 | 14.4 ± 0.2^e^ | 7.8 ± 0.4 |
| *Auricularia auricula-judae* | Jelly ear | 96.7 ± 3.3 | 71.4 ± 0.5 | 28.6 ± 0.5 | 4.1 ± 0.0^e^ | 4.7 ± 0.4^e^ | 12.8 ± 0.2^e^ | 7.1 ± 0.2 |
| *Boletus edulis* | Porcini mushroom | 98.0 ± 2.4 | 76.6 ± 0.0 | 23.4 ± 0.0 | 3.0 ± 0.2 | 1.3 ± 0.1^e^ | 14.8 ± 0.3^e^ | 4.4 ± 0.1^e^ |
| *Cantharellus cibarius* | Golden chanterelle | 99.3 ± 0.1 | 74.5 ± 0.3 | 25.5 ± 0.3 | 2.7 ± 0.1 | 2.3 ± 0.4^e^ | 14.7 ± 0.3^e^ | 5.7 ± 0.2 |
| *Craterellus cornucopioides* | Black trumpet | 98.9 ± 0.7 | 72.9 ± 0.5 | 27.1 ± 0.5 | 3.0 ± 0.0 | 2.4 ± 0.1^e^ | 15.3 ± 0.4^e^ | 6.3 ± 0.3 |
| *Flammulina velutipes* | Winter mushroom | 93.8 ± 2.0 | 76.6 ± 0.4 | 23.4 ± 0.4 | 3.5 ± 0.2 | 3.4 ± 0.3 | 9.8 ± 0.3^e^ | 6.8 ± 0.4 |
| *Grifola frondosa* | Maitake mushroom | 95.7 ± 2.7 | 75.1 ± 0.3 | 24.9 ± 0.3 | 3.4 ± 0.1 | 3.5 ± 0.0 | 11.4 ± 0.1^e^ | 6.5 ± 0.4 |
| *Hericium erinaceum* | Monkey head mushroom | 96.9 ± 1.8 | 72.4 ± 0.3 | 27.6 ± 0.3 | 3.0 ± 0.2 | 3.2 ± 0.1 | 14.9 ± 0.2^e^ | 6.6 ± 0.3 |
| *Hypsizygus marmoreus* | Buna shimeji | 99.5 ± 2.7 | 75.0 ± 0.6 | 25.0 ± 0.6 | 3.2 ± 0.1 | 2.9 ± 0.0 | 12.1 ± 0.0^e^ | 6.8 ± 0.6 |
| *Lentinula edodes* | Shiitake mushroom | 99.3 ± 0.6 | 55.8 ± 0.2^e^ | 44.2 ± 0.2^e^ | 8.3 ± 0.1^e^ | 7.7 ± 0.1^e^ | 9.4 ± 0.7^e^ | 18.9 ± 0.7^e^ |
| *Lepista nuda* | Wood Blewit | 97.4 ± 1.0 | 70.2 ± 0.5 | 29.8 ± 0.5 | 3.0 ± 0.1 | 2.4 ± 0.2^e^ | 17.7 ± 0.2^e^ | 6.8 ± 0.3 |
| *Lyophyllum shimeji* | Shimeji | 98.0 ± 1.3 | 77.6 ± 0.1 | 22.4 ± 0.1 | 2.9 ± 0.1 | 2.4 ± 0.3^e^ | 11.5 ± 0.1^e^ | 5.5 ± 0.3 |
| *Pholiota nameko* | Nameko | 92.6 ± 0.6 | 72.0 ± 0.7 | 28.0 ± 0.7 | 3.2 ± 0.0 | 3.1 ± 0.3 | 13.9 ± 0.3^e^ | 7.8 ± 0.7 |
| *Pleurotus abalonus* | Abalone mushroom | 95.8 ± 4.5 | 72.7 ± 1.3 | 27.3 ± 1.3 | 3.0 ± 0.3 | 2.9 ± 0.1 | 14.3 ± 0.3^e^ | 7.1 ± 1.0 |
| *Pleurotus citrinopileatus* | golden oyster mushroom | 93.3 ± 2.4 | 75.0 ± 0.3 | 25.0 ± 0.3 | 3.2 ± 0.2 | 3.5 ± 0.1 | 11.2 ± 0.2^e^ | 7.0 ± 0.5 |
| *Pleurotus eryngii* | King oyster mushroom | 94.4 ± 2.0 | 70.7 ± 0.3 | 29.3 ± 0.3 | 3.7 ± 0.1^e^ | 3.8 ± 0.1 | 14.0 ± 0.0^e^ | 7.7 ± 0.3 |
| *Pleurotus ostreatus* | Oyster mushroom | 94.1 ± 1.9 | 70.7 ± 0.5 | 29.3 ± 0.5 | 2.9 ± 0.6 | 3.6 ± 0.1 | 14.6 ± 0.2^e^ | 8.2 ± 0.1 |
| *Sparassis crispa* | Cauliflower Mushroom | 96.3 ± 3.5 | 68.5 ± 0.3 | 31.5 ± 0.3 | 3.5 ± 0.4 | 4.8 ± 0.3^e^ | 14.9 ± 0.3^e^ | 8.2 ± 0.6 |
| *Tricholoma matsutake* | Matsutake mushroom | 98.7 ± 1.5 | 71.7 ± 0.7 | 28.3 ± 0.7 | 3.1 ± 0.1 | 3.2 ± 0.2 | 14.5 ± 0.3^e^ | 7.5 ± 0.4 |
| *Tuber aestivum* | Summer truffle | 96.1 ± 4.0 | 74.3 ± 1.3 | 25.7 ± 1.3 | 3.2 ± 0.2 | 3.5 ± 0.8 | 11.9 ± 0.1^e^ | 7.2 ± 0.3 |

The thermal treatment of the mixture of tomato puree, fresh mushroom, and olive oil (60:35:5, w/w) was carried out at 80 °C for 1 h.

^a^Remaining ratio of total amount of lycopene isomers without decomposition by the heat treatment.

^b^Percentage content of *Z*-isomers of lycopene relative to the total amount of lycopene.

^c^Total content of *Z*-isomers of lycopene.

^d^Sum of *Z*-isomers of lycopene other than 5*Z*-, 9*Z*-, and 13*Z*-forms.

^e^Significant, *p* < 0.05 versus control (*μ*_0_ ≠ *μ*_i_).

**Table S4. Effect of various dried spices and herbs on the thermal *E*/*Z*-isomerization of lycopene contained in tomato puree**

| Scientific name | General name | Remaining lycopene^a^ | Content (%)^b^ | | | | | |
| --- | --- | --- | --- | --- | --- | --- | --- | --- |
|  |  |  | (All-*E*) | Total *Z*^c^ | (5*Z*) | (9*Z*) | (13*Z*) | Other *Z*^d^ |
| (Control) | – | 97.0 ± 2.7 | 66.6 ± 0.8 | 33.4 ± 0.8 | 5.6 ± 0.4 | 5.9 ± 0.2 | 14.4 ± 0.1 | 7.6 ± 0.1 |
| *Allium cepa* | Onion | 98.1 ± 1.3 | 34.3 ± 1.1^e^ | 65.7 ± 1.1^e^ | 14.6 ± 0.2^e^ | 11.0 ± 0.2^e^ | 9.4 ± 0.4^e^ | 30.7 ± 0.4^e^ |
| *Allium sativum* | Garlic | 98.5 ± 1.7 | 34.1 ± 0.2^e^ | 65.9 ± 0.2^e^ | 12.9 ± 0.1^e^ | 7.8 ± 0.1^e^ | 8.3 ± 0.1^e^ | 36.9 ± 0.2^e^ |
| *Brassica juncea* | Mustard | 99.0 ± 0.6 | 51.4 ± 1.2^e^ | 48.6 ± 1.2^e^ | 9.3 ± 0.2^e^ | 8.6 ± 0.5^e^ | 10.4 ± 0.2^e^ | 20.4 ± 1.5^e^ |
| *Capsicum chinense* | Habanero pepper | 95.3 ± 2.0 | 78.0 ± 0.1^e^ | 22.0 ± 0.1^e^ | 3.5 ± 0.1^e^ | 2.8 ± 0.1^e^ | 10.2 ± 0.1^e^ | 5.5 ± 0.2^e^ |
| *Carum carvi* | Caraway | 97.5 ± 1.6 | 71.8 ± 0.1^e^ | 28.2 ± 0.1^e^ | 3.3 ± 0.1^e^ | 3.1 ± 0.1^e^ | 14.5 ± 0.3 | 7.4 ± 0.2 |
| *Cinnamomum verum* | Cinnamon | 96.7 ± 2.0 | 71.9 ± 0.4^e^ | 28.1 ± 0.4^e^ | 3.6 ± 0.1^e^ | 4.5 ± 0.6^e^ | 13.1 ± 0.8 | 6.8 ± 0.5 |
| *Cuminum cyminum* | Cumin | 95.0 ± 3.3 | 59.1 ± 0.5^e^ | 40.9 ± 0.5^e^ | 9.5 ± 0.2^e^ | 10.2 ± 0.2^e^ | 6.8 ± 0.0^e^ | 14.5 ± 0.6^e^ |
| *Curcuma longa* | Turmeric | 96.9 ± 0.9 | 73.5 ± 0.6^e^ | 26.5 ± 0.6^e^ | 3.5 ± 0.1^e^ | 4.8 ± 0.4 | 11.6 ± 0.6^e^ | 6.6 ± 0.6 |
| *Elettaria cardamomum* | Cardamom | 98.2 ± 0.9 | 67.7 ± 0.5 | 32.3 ± 0.5 | 3.6 ± 0.0^e^ | 3.6 ± 0.1^e^ | 18.8 ± 0.1^e^ | 6.3 ± 0.6 |
| *Foeniculum vulgare* | Fennel | 99.2 ± 1.2 | 81.6 ± 0.1^e^ | 18.4 ± 0.1^e^ | 3.2 ± 0.1^e^ | 2.4 ± 0.1^e^ | 8.6 ± 0.2^e^ | 4.2 ± 0.0^e^ |
| *Laurus nobilis* | Laurel | 98.5 ± 2.0 | 70.1 ± 2.2^e^ | 29.9 ± 2.2^e^ | 6.5 ± 0.0^e^ | 10.9 ± 1.0^e^ | 7.9 ± 0.6^e^ | 4.6 ± 0.5^e^ |
| *Lepidium meyenii* | Maca | 98.7 ± 1.5 | 41.7 ± 0.4^e^ | 58.3 ± 0.4^e^ | 11.9 ± 0.0^e^ | 8.6 ± 0.1^e^ | 8.1 ± 0.1^e^ | 29.7 ± 0.5^e^ |
| *Myristica fragrans* | Nutmeg | 99.5 ± 1.0 | 68.1 ± 0.2 | 31.9 ± 0.2 | 3.1 ± 0.1^e^ | 2.8 ± 0.1^e^ | 17.8 ± 0.2^e^ | 8.2 ± 0.3 |
| *Pimenta dioica* | Allspice | 97.3 ± 1.5 | 76.8 ± 0.7^e^ | 23.2 ± 0.7^e^ | 3.3 ± 0.1^e^ | 4.4 ± 0.5^e^ | 12.0 ± 1.2^e^ | 3.5 ± 0.7^e^ |
| *Piper longum* | Long pepper | 97.7 ± 1.4 | 73.4 ± 0.3^e^ | 26.6 ± 0.3^e^ | 2.8 ± 0.0^e^ | 2.6 ± 0.1^e^ | 15.0 ± 0.4 | 6.2 ± 0.4 |
| *Piper nigrum* | Black pepper | 97.9 ± 1.9 | 66.8 ± 0.5 | 33.2 ± 0.5 | 3.4 ± 0.1^e^ | 3.3 ± 0.1^e^ | 19.9 ± 0.2^e^ | 6.6 ± 0.5 |
| *Piper nigrum* | White pepper | 92.8 ± 2.6 | 67.9 ± 0.8 | 32.1 ± 0.8 | 2.7 ± 0.0^e^ | 2.3 ± 0.1^e^ | 18.1 ± 0.2^e^ | 9.0 ± 0.5 |
| *Rosmarinus officinalis* | Rosemary | 98.2 ± 2.4 | 69.4 ± 0.7^e^ | 30.6 ± 0.7^e^ | 7.6 ± 0.1^e^ | 9.4 ± 0.2^e^ | 6.9 ± 1.2^e^ | 6.6 ± 0.8 |
| *Schinus Terebinthifolius* | Pink pepper | 98.6 ± 1.0 | 71.5 ± 0.2^e^ | 28.5 ± 0.2^e^ | 2.7 ± 0.0^e^ | 2.0 ± 0.1^e^ | 16.2 ± 0.3^e^ | 7.5 ± 0.2 |
| *Petroselinum crispum* | Parsley | 98.4± 4.6 | 71.6 ± 0.5^e^ | 28.4 ± 0.5^e^ | 5.8 ± 0.1 | 11.2 ± 1.0^e^ | 7.4 ± 0.2^e^ | 4.1 ± 0.4^e^ |
| *Pimpinella anisum* | Anise | 92.2 ± 1.6 | 69.8 ± 0.5^e^ | 30.2 ± 0.5^e^ | 3.3 ± 0.1^e^ | 3.0 ± 0.1^e^ | 15.1 ± 0.5 | 8.7 ± 0.2 |
| *Salvia officinalis* | Sage | 93.7 ± 1.5 | 75.0 ± 1.7^e^ | 25.0 ± 1.7^e^ | 4.1 ± 0.2^e^ | 13.5 ± 0.7^e^ | 4.0 ± 0.5^e^ | 3.4 ± 0.4^e^ |
| *Satureja hortensis* | Savory | 99.2 ± 1.5 | 70.7 ± 0.3^e^ | 29.3 ± 0.3^e^ | 6.6 ± 0.1^e^ | 9.4 ± 0.2^e^ | 7.1 ± 0.4^e^ | 6.2 ± 0.3 |
| *Syzygium aromaticum* | Clove | 99.0 ± 1.2 | 70.6 ± 0.2^e^ | 29.4 ± 0.2^e^ | 3.2 ± 0.1^e^ | 2.8 ± 0.1^e^ | 16.5 ± 0.2^e^ | 6.9 ± 0.4 |
| *Thymus vulgaris* | Thyme | 98.3 ± 1.2 | 66.9 ± 0.8 | 33.1 ± 0.8 | 4.8 ± 0.1^e^ | 6.9 ± 0.2 | 15.3 ± 0.3 | 6.1 ± 0.2^e^ |
| *Trigonella foenum-graceum* | Fenugreek | 97.7 ± 0.9 | 76.8 ± 0.7^e^ | 23.2 ± 0.7^e^ | 3.2 ± 0.1^e^ | 2.2 ± 0.1^e^ | 11.1 ± 0.3^e^ | 6.7 ± 0.3 |
| *Zanthoxylum bungeanum* | Sichuan pepper | 92.2 ± 0.2 | 72.2 ± 0.6^e^ | 27.8 ± 0.6^e^ | 3.2 ± 0.1^e^ | 3.2 ± 0.2^e^ | 14.3 ± 0.1 | 7.0 ± 0.3 |

The thermal treatment of the mixture of tomato puree, dried spice (or herb), and olive oil (90:5:5, w/w) was carried out at 80 °C for 1 h.

^a^Remaining ratio of total amount of lycopene isomers without decomposition by the heat treatment.

^b^Percentage content of *Z*-isomers of lycopene relative to the total amount of lycopene.

^c^Total content of *Z*-isomers of lycopene.

^d^Sum of *Z*-isomers of lycopene other than 5*Z*-, 9*Z*-, and 13*Z*-forms.

^e^Significant, *p* < 0.05 versus control (*μ*_0_ ≠ *μ*_i_).

**Table S5. Effect of various dried edible seaweeds on the thermal *E*/*Z*-isomerization of lycopene contained in tomato puree**

| Scientific name | General name | Remaining lycopene^a^ | Content (%)^b^ | | | | | |
| --- | --- | --- | --- | --- | --- | --- | --- | --- |
|  |  |  | (All-*E*) | Total *Z*^c^ | (5*Z*) | (9*Z*) | (13*Z*) | Other *Z*^d^ |
| (Control) | – | 97.0 ± 2.7 | 66.6 ± 0.8 | 33.4 ± 0.8 | 5.6 ± 0.4 | 5.9 ± 0.2 | 14.4 ± 0.1 | 7.6 ± 0.1 |
| *Cladosiphon okamuranus* | Mozuku | 92.5 ± 2.0 | 52.2 ± 9.2^e^ | 47.8 ± 9.2^e^ | 15.1 ± 4.1^e^ | 13.2 ± 0.4^e^ | 3.6 ± 0.1^e^ | 15.9 ± 5.5^e^ |
| *Ceratophyllum demersum* | Coontail | 95.4 ± 1.4 | 67.4 ± 0.3 | 32.6 ± 0.3 | 6.0 ± 0.1 | 11.7 ± 0.3^e^ | 7.3 ± 0.2^e^ | 7.5 ± 0.4 |
| *Ecklonia cava* | Kajime | 97.7 ± 1.9 | 59.6 ± 7.0 | 40.4 ± 7.0 | 11.9 ± 2.9^e^ | 12.2 ± 0.5^e^ | 5.1 ± 0.2^e^ | 11.1 ± 3.6 |
| *Ecklonia kurome* | Kurome | 98.0 ± 1.2 | 32.1 ± 3.3^e^ | 67.9 ± 3.3^e^ | 21.6 ± 0.9^e^ | 12.4 ± 0.1^e^ | 3.8 ± 0.1^e^ | 30.0 ± 2.5^e^ |
| *Gelidium elegans* | Tengusa | 92.0 ± 1.1 | 69.5 ± 1.5 | 30.5 ± 1.5 | 7.4 ± 0.3 | 10.1 ± 0.5^e^ | 4.9 ± 0.1^e^ | 8.1 ± 1.1 |
| *Gloiopeltis furcata* | Fukuro-funori | 93.8 ± 1.6 | 71.3 ± 0.7 | 28.7 ± 0.7 | 7.1 ± 0.1 | 9.3 ± 0.1^e^ | 5.5 ± 0.4^e^ | 6.7 ± 0.3 |
| *Gloiopeltis tenax* | Ma-funori | 93.4 ± 2.1 | 70.3 ± 0.8 | 29.7 ± 0.8 | 5.8 ± 0.0 | 8.2 ± 0.1 | 9.7 ± 0.2^e^ | 6.0 ± 0.6 |
| *Eisenia bicyclis* | Arame | 95.7 ± 0.6 | 39.3 ± 6.8^e^ | 60.7 ± 6.8^e^ | 19.7 ± 2.6^e^ | 7.3 ± 3.8 | 10.4 ± 3.5^e^ | 23.4 ± 4.5^e^ |
| *Monostroma nitidum* | Hitoegusa | 92.1 ± 1.1 | 66.0 ± 0.8 | 34.0 ± 0.8 | 7.5 ± 0.2 | 13.2 ± 0.2^e^ | 7.3 ± 0.3^e^ | 6.1 ± 0.4 |
| *Palmaria palmata* | Dulse | 95.1 ± 0.7 | 63.7 ± 0.3 | 36.3 ± 0.3 | 9.6 ± 0.2^e^ | 9.1 ± 0.3^e^ | 6.0 ± 0.1^e^ | 11.6 ± 0.3 |
| *Porphyra pseudolinearis* | Iwa-nori | 93.0 ± 1.0 | 60.0 ± 0.6 | 40.0 ± 0.6 | 11.4 ± 0.2^e^ | 12.4 ± 0.0^e^ | 7.3 ± 1.3^e^ | 8.9 ± 1.5 |
| *Saccharina angustata* | Mitsuishi-kombu | 94.2 ± 2.7 | 44.4 ± 5.3^e^ | 55.6 ± 5.3^e^ | 16.3 ± 1.0^e^ | 11.8 ± 0.2^e^ | 6.3 ± 1.2^e^ | 21.2 ± 2.9^e^ |
| *Saccharina japonica* | Ma-kombu | 98.0 ± 1.6 | 17.2 ± 0.1^e^ | 82.8 ± 0.1^e^ | 20.9 ± 0.1^e^ | 9.9 ± 0.1^e^ | 4.0 ± 0.1^e^ | 48.0 ± 0.3^e^ |
| *Saccharina sculpera* | Gagome-Kombu | 94.1 ± 1.8 | 33.5 ± 8.8^e^ | 66.5 ± 8.8^e^ | 17.7 ± 2.2^e^ | 11.4 ± 0.2^e^ | 8.2 ± 1.8^e^ | 29.2 ± 4.5^e^ |
| *Sargassum fusiforme* | Hijiki | 95.9 ± 0.8 | 39.6 ± 4.7^e^ | 60.4 ± 4.7^e^ | 20.0 ± 1.6^e^ | 12.7 ± 0.2^e^ | 3.7 ± 0.3^e^ | 24.0 ± 3.3^e^ |
| *Sargassum horneri* | Akamoku | 95.2 ± 2.1 | 65.1 ± 2.6 | 34.9 ± 2.6 | 8.1 ± 1.0 | 12.3 ± 0.1^e^ | 6.3 ± 0.6^e^ | 8.2 ± 1.3 |
| *Undaria pinnatifida* | Sea mustard | 93.8 ± 8.9 | 72.5 ± 0.4 | 27.5 ± 0.4 | 4.8 ± 0.2 | 11.5 ± 0.2^e^ | 6.3 ± 0.2^e^ | 4.9 ± 0.3 |
| *Undaria undarioides* | Hirome | 98.4 ± 1.6 | 70.3 ± 1.3 | 29.7 ± 1.3 | 6.5 ± 0.5 | 9.3 ± 0.8^e^ | 6.4 ± 0.4^e^ | 7.5 ± 0.5 |

The thermal treatment of the mixture of tomato puree, dried seaweed, and olive oil (90:5:5, w/w) was carried out at 80 °C for 1 h.

^a^Remaining ratio of total amount of lycopene isomers without decomposition by the heat treatment.

^b^Percentage content of *Z*-isomers of lycopene relative to the total amount of lycopene.

^c^Total content of *Z*-isomers of lycopene.

^d^Sum of *Z*-isomers of lycopene other than 5*Z*-, 9*Z*-, and 13*Z*-forms.

^e^Significant, *p* < 0.05 versus control (*μ*_0_ ≠ *μ*_i_).

**Table S6. Effect of various catalysts on the thermal *E*/*Z*-isomerization of lycopene contained in tomato puree**

| Compound | Concentration (mM) | Remaining lycopene^a^ | Content (%)^b^ | | | | | |
| --- | --- | --- | --- | --- | --- | --- | --- | --- |
|  |  |  | (All-*E*) | Total *Z*^c^ | (5*Z*) | (9*Z*) | (13*Z*) | Other *Z*^d^ |
| (Control) | – | 96.6 ± 2.7 | 69.5 ± 0.6 | 30.5 ± 0.6 | 3.0 ± 0.2 | 3.5 ± 0.2 | 16.6 ± 0.1 | 7.4 ± 0.4 |
| Diallyl disulfide | 0.01 | 96.1 ± 3.0 | 65.1 ± 0.4 | 34.9 ± 0.4 | 4.4 ± 0.3 | 5.0 ± 0.2^e^ | 14.8 ± 0.1^e^ | 10.8 ± 0.0 |
|  | 0.1 | 98.4 ± 0.3 | 63.4 ± 0.4^e^ | 36.6 ± 0.4^e^ | 5.2 ± 0.1^e^ | 6.0 ± 0.0^e^ | 12.9 ± 0.2^e^ | 12.4 ± 0.4 |
|  | 1 | 96.3 ± 2.4 | 47.5 ± 0.8^e^ | 52.5 ± 0.8^e^ | 10.7 ± 0.3^e^ | 10.1 ± 0.3^e^ | 7.9 ± 0.3^e^ | 23.7 ± 0.5^e^ |
|  | 10 | 99.2 ± 0.7 | 34.4 ± 0.6^e^ | 65.7 ± 0.6^e^ | 15.7 ± 0.1^e^ | 9.7 ± 0.2^e^ | 5.9 ± 0.2^e^ | 34.4 ± 0.5^e^ |
| Diallyl trisulfide | 0.01 | 95.7 ± 0.7 | 62.1 ± 0.6^e^ | 37.9 ± 0.6^e^ | 5.7 ± 0.3^e^ | 7.6 ± 0.8^e^ | 16.1 ± 2.5 | 8.5 ± 3.8 |
|  | 0.1 | 97.2 ± 1.1 | 45.4 ± 0.4^e^ | 54.6 ± 0.4^e^ | 11.3 ± 0.0^e^ | 9.5 ± 0.1^e^ | 8.3 ± 0.2^e^ | 25.5 ± 0.4^e^ |
|  | 1 | 98.7 ± 0.8 | 35.2 ± 0.7^e^ | 64.8 ± 0.7^e^ | 13.9 ± 0.1^e^ | 8.0 ± 0.0^e^ | 7.9 ± 0.2^e^ | 35.0 ± 0.5^e^ |
|  | 10 | 95.5 ± 1.1 | 30.5 ± 0.3^e^ | 69.5 ± 0.3^e^ | 15.4 ± 0.0^e^ | 4.6 ± 0.1 | 8.9 ± 0.1^e^ | 40.7 ± 0.2^e^ |
| Lenthionine | 0.01 | 98.8 ± 1.1 | 48.5 ± 0.6^e^ | 51.5 ± 0.6^e^ | 8.7 ± 0.1^e^ | 8.6 ± 0.2^e^ | 11.3 ± 0.1^e^ | 22.9 ± 0.4^e^ |
|  | 0.1 | 98.8 ± 1.3 | 37.7 ± 0.5^e^ | 62.3 ± 0.5^e^ | 12.2 ± 0.3^e^ | 9.5 ± 0.1^e^ | 10.0 ± 0.5^e^ | 30.7 ± 0.6^e^ |
|  | 1 | 97.9 ± 1.6 | 30.5 ± 0.4^e^ | 69.5 ± 0.4^e^ | 14.4 ± 0.1^e^ | 10.0 ± 0.2^e^ | 8.6 ± 0.2^e^ | 36.6 ± 0.3^e^ |
|  | 10 | 99.5 ± 0.4 | 24.8 ± 0.9^e^ | 75.2 ± 0.9^e^ | 15.9 ± 0.1^e^ | 12.1 ± 1.0^e^ | 9.0 ± 0.1^e^ | 38.1 ± 1.7^e^ |
| Allyl isothiocyanate | 0.01 | 98.0 ± 1.4 | 65.2 ± 0.7 | 34.8 ± 0.7 | 4.4 ± 0.3 | 5.0 ± 0.1^e^ | 15.2 ± 0.1 | 10.2 ± 0.5 |
|  | 0.1 | 96.3 ± 2.3 | 51.1 ± 0.5^e^ | 48.9 ± 0.5^e^ | 8.4 ± 0.1^e^ | 8.6 ± 0.1^e^ | 10.6 ± 0.4^e^ | 21.3 ± 0.7^e^ |
|  | 1 | 98.1 ± 2.0 | 41.4 ± 0.8^e^ | 58.6 ± 0.8^e^ | 13.2 ± 0.2^e^ | 8.9 ± 0.1^e^ | 7.3 ± 0.3^e^ | 29.2 ± 1.1^e^ |
|  | 10 | 98.8 ± 0.3 | 31.6 ± 0.9^e^ | 68.4 ± 0.9^e^ | 16.0 ± 0.3^e^ | 9.2 ± 0.2^e^ | 6.1 ± 0.3^e^ | 37.0 ± 0.6^e^ |
| Benzyl isothiocyanate | 0.01 | 99.1 ± 0.5 | 64.8 ± 0.3 | 35.2 ± 0.3 | 4.6 ± 0.1 | 5.1 ± 0.3^e^ | 14.9 ± 0.1^e^ | 10.6 ± 0.3 |
|  | 0.1 | 95.5 ± 1.5 | 63.3 ± 0.3^e^ | 36.7 ± 0.5^e^ | 4.5 ± 0.2 | 4.7 ± 0.2^e^ | 15.9 ± 0.1 | 11.5 ± 0.2 |
|  | 1 | 99.4 ± 0.6 | 54.0 ± 0.8^e^ | 46.0 ± 0.8^e^ | 8.3 ± 0.3^e^ | 8.5 ± 0.2^e^ | 10.1 ± 0.1^e^ | 19.0 ± 0.6^e^ |
|  | 10 | 98.6 ± 1.6 | 34.6 ± 0.8^e^ | 65.4 ± 0.8^e^ | 14.3 ± 0.2^e^ | 8.6 ± 0.2^e^ | 6.4 ± 0.1^e^ | 36.1 ± 0.5^e^ |
| Carbon disulfide | 0.01 | 97.4 ± 1.1 | 61.4 ± 1.9^e^ | 38.6 ± 1.9^e^ | 6.1 ± 0.8^e^ | 6.5 ± 0.4^e^ | 14.0 ± 0.2^e^ | 12.0 ± 0.9 |
|  | 0.1 | 97.7 ± 1.5 | 59.9 ± 0.7^e^ | 40.1 ± 0.7^e^ | 4.6 ± 0.1 | 5.7 ± 0.1^e^ | 16.9 ± 0.2 | 12.9 ± 0.3^e^ |
|  | 1 | 98.7 ± 1.4 | 46.2 ± 0.3^e^ | 53.8 ± 0.3^e^ | 9.8 ± 0.1^e^ | 8.8 ± 0.2^e^ | 10.2 ± 0.2^e^ | 24.9 ± 0.1^e^ |
|  | 10 | 92.6 ± 1.8 | 29.3 ± 1.3^e^ | 70.7 ± 1.3^e^ | 14.3 ± 0.4^e^ | 10.7 ± 0. 2^e^ | 8.6 ± 0.1^e^ | 37.0 ± 0.8^e^ |
| Iodine | 0.01 | 98.4 ± 0.5 | 57.3 ± 2.2^e^ | 42.7 ± 2.2^e^ | 7.1 ± 0.9^e^ | 5.8 ± 0.5^e^ | 14.4 ± 0.2^e^ | 15.4 ± 1.0^e^ |
|  | 0.1 | 97.2 ± 0.1 | 35.0 ± 8.2^e^ | 65.5 ± 8.2^e^ | 15.2 ± 2.2^e^ | 9.4 ± 0.3^e^ | 8.5 ± 0.4^e^ | 32.0 ± 6.1^e^ |
|  | 1 | 94.8 ± 1.8 | 21.4 ± 3.9^e^ | 78.6 ± 3.9^e^ | 18.4 ± 0.8^e^ | 9.9 ± 0.2^e^ | 7.4 ± 0.8^e^ | 43.0 ± 3.9^e^ |
|  | 10 | –^f^ | – | – | – | – | – | – |

The thermal treatment of the mixture of tomato puree, water, and olive oil (60:35:5, w/w) containing defined amount of catalysts was carried out at 80 °C for 1 h.

^a^Remaining ratio of total amount of lycopene isomers without decomposition by the heat treatment.

^b^Percentage content of *Z*-isomers of lycopene relative to the total amount of lycopene.

^c^Total content of *Z*-isomers of lycopene.

^d^Sum of *Z*-isomers of lycopene other than 5*Z*-, 9*Z*-, and 13*Z*-forms.

^e^Significant, *p* < 0.05 versus control (*μ*_0_ ≠ *μ*_i_).

^f^All lycopene was decomposed after the heat treatment.

**Figure S1. Contents of lycopene *Z*-isomers in commercially available raw tomatoes and processed tomato foods.** **1**, Made by Kagome Co., Ltd.^9^; **2**, Made by Feel Co., Ltd.^9^; **3**, Made by Ito en Ltd.^9^; **4**, Made by Fresh Del Monte Japan Co., Ltd.^9^; **5**, Made by Heinz & Company Co., Ltd.^9^; **6**, Made by Taiyo Shokuhin Kogyo Co., Ltd.; **7**, Made by Kagome Co., Ltd.; **8**, Made by Nagano Tomato Co., Ltd.; **9**, Made by Nisshin Seifun Group Inc.; **10**, Made by Kisai Foods Kogyo Co., Ltd.; **11**, Made by Ryohin Keikaku Co., Ltd.; **12**, Made by Miyajima Shoyu Co., Ltd.; **13**, Made by Naniwa Kombu Co., Ltd. Error bars show standard deviation (*n* = 2 or 3). The exact thermal history (sterilization condition), the content of each food material, and the lycopene content of the products are unknown.


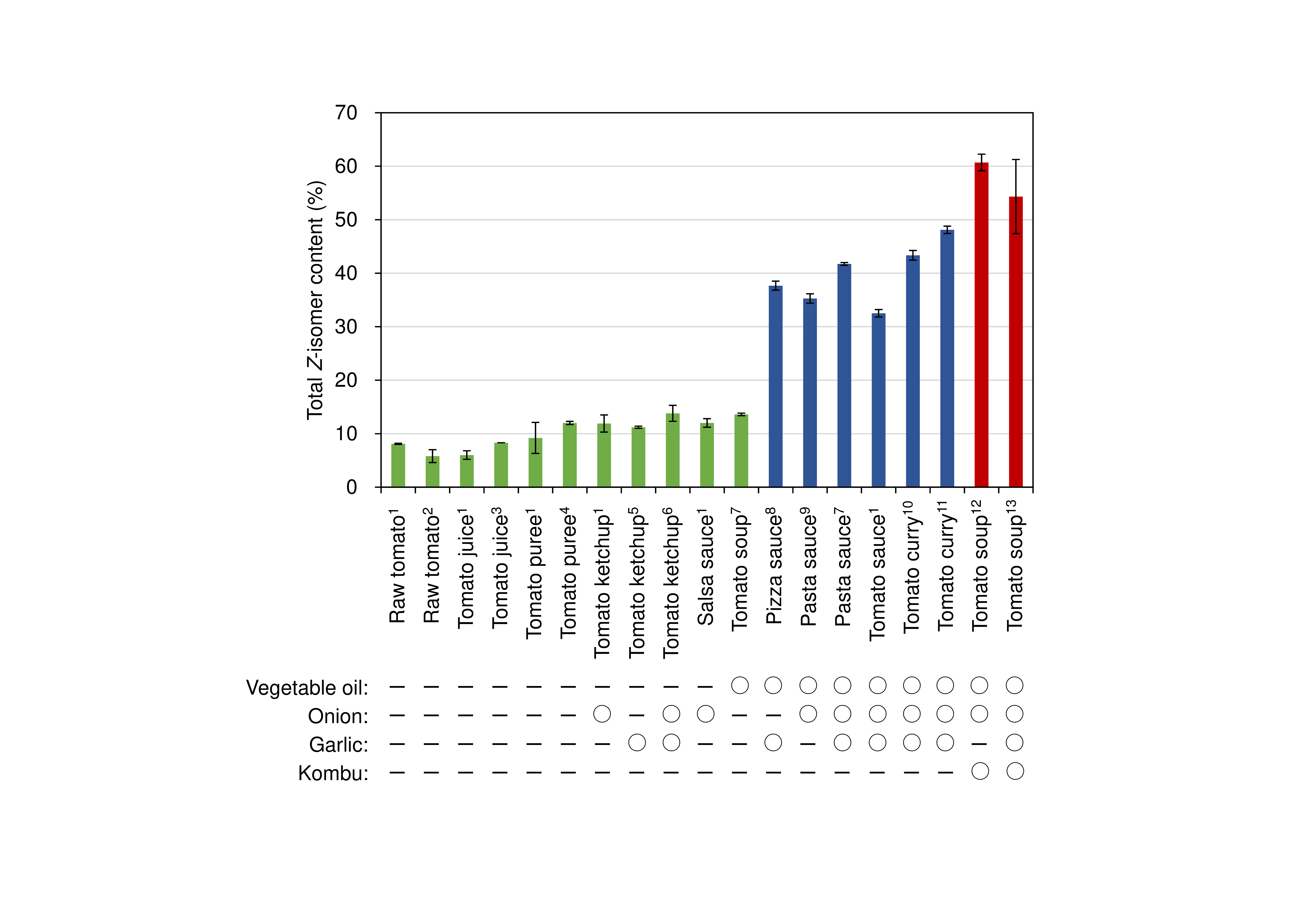


**Figure S2. Increase in the efficiency of carotenoid processing utilizing food-derived and high-volatility *Z*-isomerization promoting catalysts. a**, General carotenoid processing procedure. Most carotenoids are present in the all-*E*-configuration in plants and microalgae. As (all-*E*)-carotenoids exhibit low solubility in solvents and high crystallinity, processing efficiencies such as extraction, micronization, and emulsification are low^39–41^. **b**, Proposed new carotenoid processing utilizing food-derived and high-volatility *Z*-isomerization promoting catalysts. Polysulfides, isothiocyanates, and carbon disulfide found in this study could be used as catalysts. It is suggested that by adding the catalysts to the extraction step, the *Z*-isomerization of carotenoids is promoted, the solubility increased, and thus the extraction efficiency is improved^38,39^. Since the catalysts exhibit relatively low volatility, they could be removed via a solvent removal step. As the obtained extract would contain a large amount of carotenoid *Z*-isomers exhibiting high solubility and low crystallinity, it has high processability, i.e., when using an extract rich in carotenoid *Z*-isomers, the processing efficiencies of micronization, emulsification, and microencapsulation would be higher compared to using an extract rich in (all-*E*)-carotenoids^40,41^. Moreover, supplements, food colorants, and cosmetics containing carotenoids rich in *Z*-isomers would have higher bioavailability^5,6^ and antioxidant capacity^11^ compared to those rich in the all-*E*-isomer.


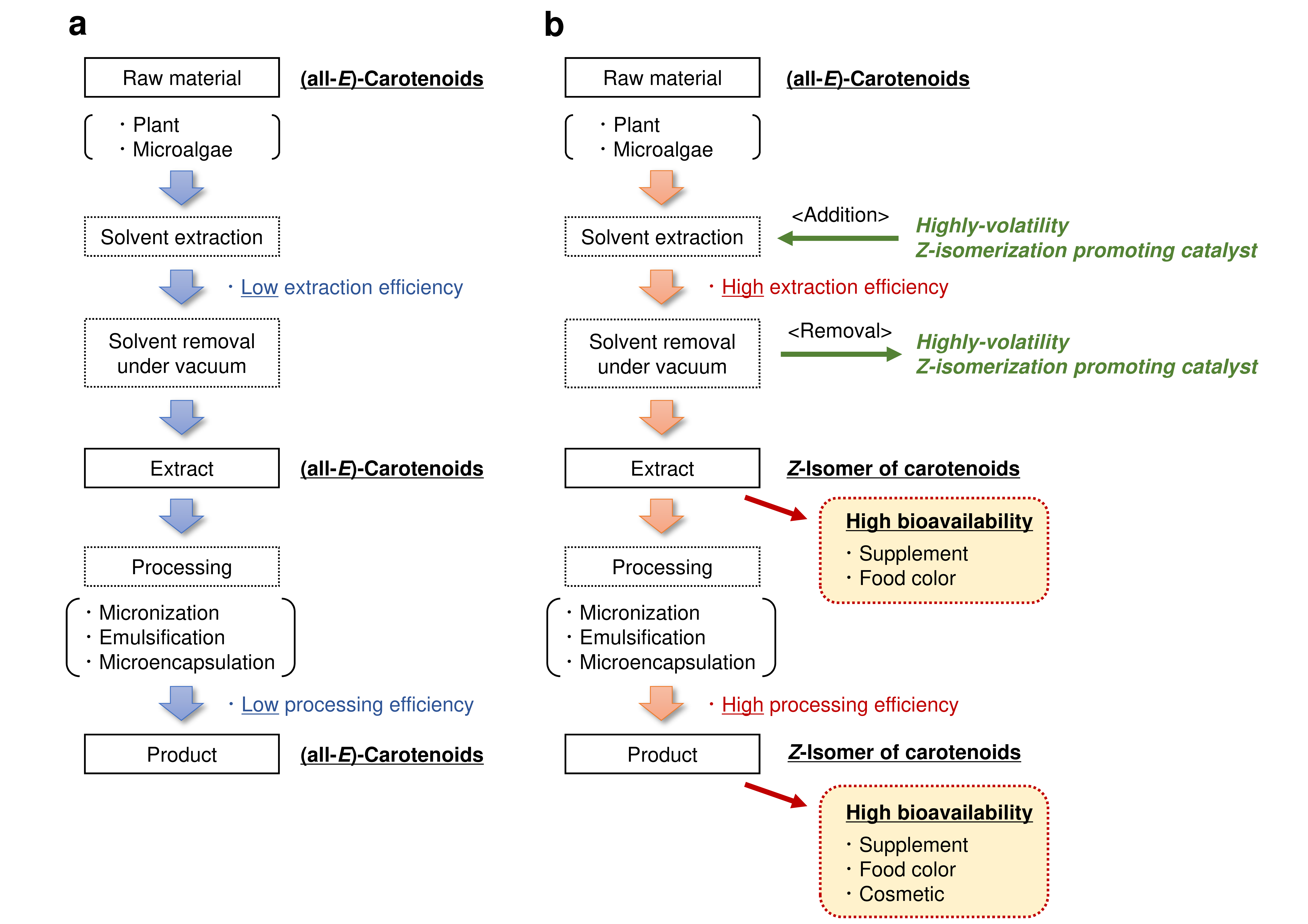

Supplement: Supplementary file 1 — Table S1, Table S2, Table S3, Table S4, Table S5, Table S6, Figure S1, Figure S2 [file 41598_2019_44177_MOESM1_ESM.docx]
